# Supplementary material for: Effect of HHP, Enzymes and Gelatin on Physicochemical Factors of Gels Made by Using Protein Isolated from Common Cricket (Acheta domesticus)
Source: Foods. 2021 Apr 15;10(4):858. doi: 10.3390/foods10040858 (PMC8071182; doi:10.3390/foods10040858)
Supplement: Supplementary file 1 [file foods-10-00858-s001.zip › foods-1175952-supplementary.pdf]

foods

MDPI

ANNEX

Data availability and supplementary material

Table S1. Hardness histogram pellet.

EA-P_7%	EA-S_7%_SD	EA-P_11%	EA-P_11%_S	EA-P_15%	EA-P_15%_S	EB-P_7%	EB-P_7%_SD	EB-P_11%	EB-P_11%_S	EB-P_15%	EB-P_15%_S	WPI_7%	WPI_7%_SD	WPI_11%	WPI_11%_SD	WPI_15%	WPI_15%_SD
31,69	10,2	44,83	13,72	41,41	10,71	27,69	5,96	36,85	11,77	43,77	5,23	37,02	5,17	46,32	2,58	51,27	2,37
107,8	20	121,98	25	112,73	30	105,19	29	123	32	120,55	35	65,21	15,56	95,71	14,76	112,53	6,47
92,16	13,922	179,69	20	241,78	16,209	105,05	14,964	194,45	25	168,46	13,9	131,79	7,61	160,64	5	158,78	9,81

Table S2. Hardness histogram soluble.

EA-S_7%	EA-S_7%_SD	EA-S_11%	EA-S_11%_S	EA-S_15%	EA-S_15%_S	EB-S_7%	EB-S_7%_SD	EB-S_11%	EB-S_11%_S	EB-S_15%	EB-S_15%_S	WPI_7%	WPI_7%_SD	WPI_11%	WPI_11%_SD	WPI_15%	WPI_15%_SD
27,62	2,03	32,03	2,47	35,75	5,43	37,62	4,5	37,2	3,84	41,03	3,13	37,02	5,17	46,32	2,58	51,27	2,37
79,36	10	77,06	15	87,68	12	82,34	5,54	91,27	16	130,62	15	65,21	8	95,71	14,76	112,53	6,47
73,4	15	80,82	16	70,62	20	90,15	23	85,79	20	140,8	10,84	131,79	7,61	160,64	5	158,78	9,81

Table S3. Syneresis histogram pellet.

EA-P_7%	EA-P_SD_7%	EA-P_11%	EA-P_11%_S	EA-P_15%	EA-P_15%_S	EB-P_7%	EB-P_7%_SD	EB-P_11%	EB-P_11%_S	EB-P_15%	EB-P_15%_S	WPI_7%	WPI_7%_SD	WPI_11%	WPI_11%_SD	WPI_15%	WPI_15%_SD
6,8	0,23	12,29	0,18	2,68	0,25	12,29	0,25	2,68	0,4	3,16	0,3	14,06	1,5	6,57	2,5	6,84	1,8
12,38	0,16	13,36	0,19	10,69	0,16	13,36	0,1	10,69	0,25	5,95	0,2	45,69	2	14,21	2	15,98	2,4
7,26	0,2	10,2	0,4	4,1	0,2	10,2	0,3	4,1	0,19	2,27	0,18	19	3	10	3	12	2,7

Table S4. Syneresis histogram soluble.

EA-S_7%	EA_S_SD_7%	EA-S_11%	EA_S_11%_S	EA-S_15%	EA_S_15%_S	EB-S_7%	EB_S_7%_SD	EB-S_11%	EB_S_11%_S	EB-S_15%	EB_S_15%_S	WPI_7%	WPI_7%_SD	WPI_11%	WPI_11%_SD	WPI_15%	WPI_15%_SD
19,55	0,3	17,08	0,2	15,41	0,25	9,41	0,2	8,12	0,35	8,21	0,2	14,06	1,5	6,57	2,5	6,84	1,8
20,08	0,2	17,78	0,3	14,97	0,15	12,27	0,25	11,26	0,2	13,48	0,3	45,69	2	14,21	2	15,98	2,4
19,63	0,23	21,15	0,1	11,43	0,4	6,89	0,3	7,97	0,25	10,77	0,18	19	3	10	3	12	2,7

Table S5. Figure of pH evolution pellet, acid and alkaline extraction.

DAYS	EA_P 7% SD		EA_P 11% SD		EA_P 15% SD		EB_P 7% SD		EB_P 11% SD		EB_P 15% SD	
1	5,72	0,03	5,58	0,06	5,71	0,05	8,19	0,06	8,6	0,05	8,4	0,03
7	5,61	0,05	5,64	0,05	5,64	0,04	8,13	0,07	8,39	0,07	8,42	0,05
14	5,6	0,06	5,64	0,03	5,71	0,03	8,21	0,03	8,33	0,06	8,42	0,06
21	5,6	0,04	5,44	0,04	5,71	0,04	7,96	0,04	8,4	0,04	8,32	0,04
28	5,54	0,03	5,6	0,05	5,76	0,03	8,56	0,06	8,23	0,03	8,23	0,07

Table S6. Figure of pH evolution soluble, acid and alkaline extraction.

DAYS	EA_S 7% SD		EA_S 11% SD		EA_S 15% SD		EB_S 7% SD		EB_S 11% SD		EB_S 15% SD	
1	6,22	0,02	6,1	0,05	6,04	0,04	8,48	0,02	8,74	0,05	8,81	0,06
7	6,2	0,03	6,04	0,03	6	0,03	8,51	0,03	8,74	0,03	8,83	0,03
14	6,15	0,04	6,03	0,02	6,1	0,04	8,56	0,05	8,74	0,05	8,81	0,04
21	6,17	0,02	6,23	0,02	6,04	0,05	8,45	0,02	8,67	0,02	8,73	0,04
28	5,97	0,03	5,99	0,03	5,94	0,03	8,34	0,03	8,35	0,03	8,65	0,07

Table S7. Figure of pH evolution WPI.

DAYS	WPI 7% SD		WPI 11% SD		WPI 15% SD	
1	6,24	0,02	6,27	0,04	6,23	0,05
7	6,22	0,04	6,2	0,02	6,28	0,02
14	6,11	0,03	6,55	0,05	6,26	0,04
21	6,32	0,02	6,41	0,02	6,26	0,02
28	6,15	0,03	6,21	0,03	6,23	0,03

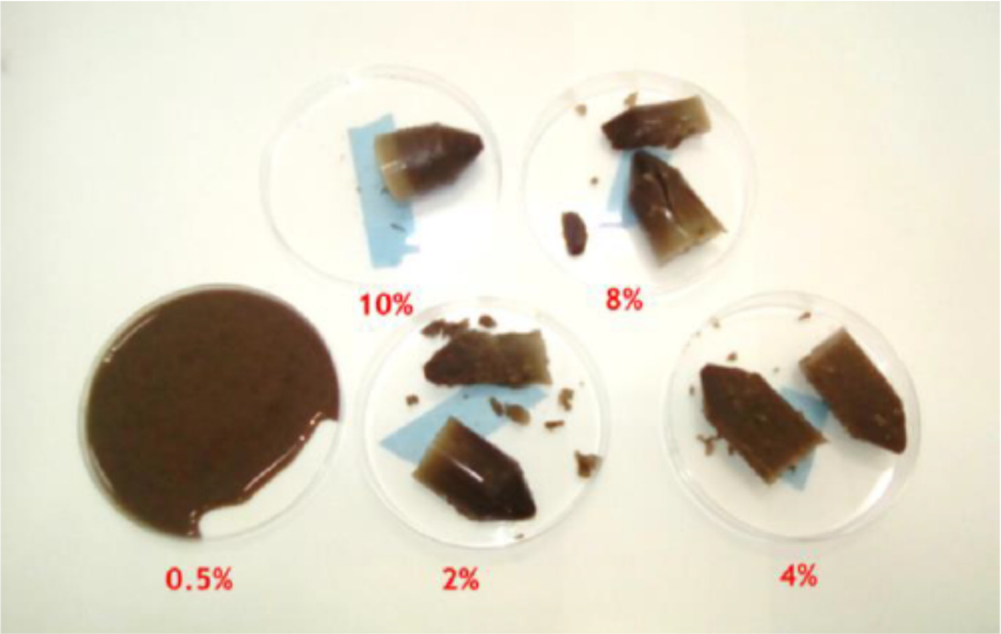

Figure S1. Gelatine and insect protein isolated gels.

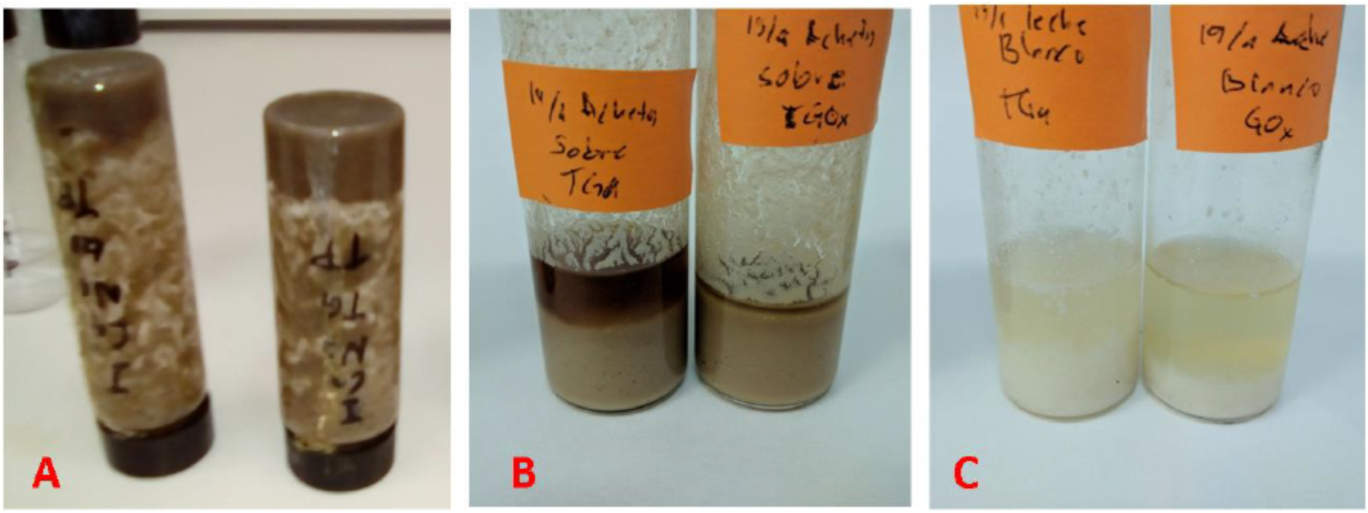

Figure S2. Test tubes after incubation with enzymes **A**) Pasty pellet fractions **B**) Viscous supernatant fractions **C**) WPI forming clots.
